# Dietary patterns and associations with biomarkers of inflammation in adults: a systematic review of observational studies

**DOI:** 10.1186/s12937-021-00674-9

**Published:** 2021-03-12

**Authors:** Michael J. Hart, Susan J. Torres, Sarah A. McNaughton, Catherine M. Milte

**Affiliations:** Institute for Physical Activity and Nutrition, School of Exercise and Nutrition Sciences, Deakin University, Geelong, VIC 3220 Australia

**Keywords:** Systematic review, Dietary patterns, Dietary intake, Inflammation, Biomarker, C-reactive protein, CRP, Interleukin

## Abstract

**Background:**

Evidence indicates that low-grade inflammation is involved in manychronic diseases of ageing. Modifiable lifestyle factors including dietcan affect low-grade inflammation. Dietary patterns allow assessment of the complex interactions of food nutrients and health and may be associated with inflammatory status.

This systematic review aimed to summarises current evidence from observational studies for associations between dietary patterns and inflammatory biomarkers in the general adult population. This review followed the PRISMA guidelines.

**Methods:**

We conducted a systematic search in Embase, CINAHL Complete, Global Health and MEDLINE complete databases. Search terms included terms for diet (“dietary patterns”, “diet scores”) and inflammation (“inflammation“, “c-reactive protein“, “interleukin“).

**Results:**

The search produced 7161 records. Duplicates were removed leaving 3164 for screening. There were 69 studies included (60 cross-sectional, 9 longitudinal). Papers included studies that were: 1) observational studies; 2) conducted in community-dwelling adults over 18 years of age; 3) assessed dietary patterns; 4) measured specified biomarkers of inflammation and 5) published in English. Dietary patterns were assessed using diet scores (*n* = 45), data-driven approaches (*n* = 22), both a data-driven approach and diet score (*n* = 2). The most frequently assessed biomarkers were CRP (*n* = 64) and/or IL-6 (*n* = 22). Cross-sectionally the majority of analyses reported an association between higher diet scores (mostly Mediterranean and anti-inflammatory diet scores) and lower inflammatory markers with 82 significant associations from 133 analyses. Only 22 of 145 cross-sectional analyses using data-driven approaches reported an association between a dietary patterns and lower inflammatory markers; the majority reported no association. Evidence of an association between dietary patterns and inflammatory markers longitudinally is limited, with the majority reporting no association.

**Conclusions:**

Adherence to healthy, Mediterranean and anti-inflammatory dietary scores, appear to be associated with lower inflammatory status cross-sectionally. Future research could focus on longitudinal studies using a potential outcomes approach in the data analysis.

**Trial registration:**

PROSPERO Registration Number CRD42019114501.

**Supplementary Information:**

The online version contains supplementary material available at 10.1186/s12937-021-00674-9.

## Introduction

Inflammation is an essential physiological response for maintaining health and recovering from injury. There is also a growing body of evidence indicating that chronic low-grade inflammation, in the absence of a trigger of acute inflammation, is part of the aetiology of many of the chronic diseases of ageing [1]. Inflammation of the endothelium has been associated with cardiovascular disease (CVD) and hypertension, and to the development of insulin resistance, type 2 diabetes (T2D) [2] and 15% of cancers [3], including bladder, oesophageal, prostate and thyroid [4]. There is also evidence that neuroinflammation may contribute to the development of depression, cognitive decline and dementia [5]. These diseases are a significant risk factor for both morbidity and mortality in ageing [6]. Inflammation (including low-grade inflammation) can be measured using serum inflammatory biomarkers, the levels of which provides an understanding of the inflammatory status within the body. Higher serum levels of C-reactive protein (CRP), interleukin 6 (IL-6), tumour necrosis factor α (TNF- α) and fibrinogen indicate higher levels of systemic inflammation, while lower levels of adiponectin indicate lower levels of inflammation [7–9].

Evidence has emerged that lifestyle factors can modify low-grade inflammation [10]. Pro-inflammatory lifestyle factors include inactivity, excessive exercise (overtraining), unhealthy diet, obesity, smoking, stress, and sleep deprivation. Anti-inflammatory lifestyle factors include exercise, healthy diet and weight loss or healthy weight [10, 11]. Historically, studies of the link between dietary intake and inflammation assessed individual vitamins and nutrients, for example, the anti-inflammatory potential of omega-3 fatty acids in the diet [12]. Assessing individual vitamins and nutrients, while important, does not take into account that the whole diet or “dietary pattern” which recognises that foods are consumed in complex combinations. In addition, the balance of the various factors may be more important than individual components [13]. Dietary patterns can be assessed using either a priori diet scores which are pre-defined scores based on dietary guidelines such as the Healthy Eating Index (HEI) [14, 15], existing literature such as the Dietary Inflammatory Index (DII) [16] or recognised patterns of food consumption such as the Mediterranean diet [17, 18]. Dietary patterns can also be assessed using a posteriori data-driven approaches, where the data collected is used to identify patterns of food consumption using statistical methods such as principal component analysis (PCA) and/or cluster analysis.

Existing reviews examining dietary patterns and inflammation have focussed on specific dietary patterns. For example, Schwingshackl et al. in 2014, focused on the Mediterranean diet and the inflammatory markers CRP, IL-6, adiponectin, intercellular adhesion molecule-1 and vascular adhesion molecule-1. The meta-analysis identified 17 intervention trials and (*n* = 2300) concluded the Mediterranean diet was anti-inflammatory [19]. Using weighted mean differences they found significantly decreased CRP (WMD: − 0.98 mg/l, 95% CI − 1.48 to − 0.49, *p* < 0.0001; I^2^ = 91%), adiponectin (WMD: 1.69 μg/ml, 95% CI 0.27 to 3.11, *p* = 0.02; I^2^ = 78%), IL-6 (WMD: − 0.42 pg/ml, 95% CI − 0.73 to − 0.11, *p* = 0.008; I^2^ = 81%) and intracellular adhesion molecule-1 (WMD: − 23.73 ng/ml, 95% CI − 41.24 to − 6.22 *p* = 0.008; I^2^ = 34%). A systematic review by Barbaresko et al. in 2013 [20], examining 43 cross-sectional studies of diverse dietary patterns and three intervention studies of the Mediterranean diet, concluded there was some evidence for the association between dietary patterns and inflammatory biomarkers. However, no prospective studies were identified for that review and they concluded that prospective studies were needed [20]. Given the specific focus of previous reviews and the publication of new primary studies, a comprehensive review of dietary patterns and inflammation including prospective studies is warranted. Therefore, the aim of this systematic review is to summarise the current evidence from observational studies for an association between dietary patterns and the inflammatory biomarkers adiponectin, CRP, fibrinogen, IL-6 and TNF-α in the general adult population. This systematic review followed the Preferred Reporting Items for Systematic reviews and Meta-Analyses (PRISMA) guidelines and includes the PRISMA checklist and flow diagram [21].

## .Methods

A search of the literature was performed to identify studies that assessed associations between dietary patterns (score or indices and data-driven methods) and one or more biomarker of inflammation in a general adult population aged over 18 years. Details of the protocol for this systematic review were registered on PROSPERO and can be accessed at www.crd.york.ac.uk/PROSPERO/display_record.asp?ID=CRD42019114501.

### Search strategy

The Embase, CINAHL Complete, Global Health and MEDLINE complete databases were searched for articles meeting the inclusion criteria. The literature search included free-text keywords from each of the categories: Diet (“diet* pattern*”, “food pattern*”, “diet* score*”, “diet* inde*”, “diet* indi*”, “diet* habit*”, “diet* inflammat*”, “eating inde*” “eating indi*”) and Inflammation (“inflammat*“, “c-reactive* ““crp“, “interleukin“, “il-6“, “marker* ““bio*marker*“, “tumo*r necrosis factor”, “tnf“, “adiponectin“, “fibrinogen“).

### Inclusion and exclusion criteria

Studies which met the following criteria were included in the current review (Table 1). The studies were of an observational (cross-sectional, case-control, or longitudinal cohorts), conducted in community-dwelling adults 18 years of age and over, and published in English. The studies were required to collect dietary intake data using single or multiple 24-h recall, food diaries or food frequency questionnaires (FFQ) and use these data to determine data-driven dietary patterns (eg. principal component analysis, factor analysis) or calculate a diet index or score (eg. Mediterranean Diet Score, Dietary Guidelines Index) to assess dietary intake as the exposure. The studies needed to measure one or more of the following inflammatory biomarkers as the outcome: adiponectin, CRP, fibrinogen, IL-6 or TNF- α.

**Table 1:** PICOS criteria for inclusion and exclusion of studies

Parameter	Inclusion criteria	Exclusion criteria
Population	• Community-dwelling adults aged 18 years and over • Females and males • Generally healthy population	• Clinical populations • Studies of children and adolescent populations • Studies examining pregnant or lactating women
Interventions	• Observational studies: collected data using single or multiple 24-h recall, food diaries or food frequency questionnaires and assessed diet using scores/indices or data-driven approaches including CA, FA, PCA, RRR	• Studies examining individual dietary nutrients, supplements or foods • Studies that were not observational studies
Comparisons	• Observational studies: no comparison population	
Outcomes	• One or more of the following inflammatory biomarkers: ° Adiponectin ° C-reactive protein ° Fibrinogen ° interleukin-6 ° tumour necrosis factor-alpha	• Any biomarker not listed in the inclusion criteria
Study design	• Cross-sectional studies • Case-control • Longitudinal studies	• Literature reviews • Narrative reviews • Opinion pieces • Conference abstracts • Non-study-based sources • Intervention studies • Randomized controlled trials

*CA* Cluster analysis, *FA* Factor analysis, *PCA* Principal component analysis, *RRR* Reduced-rank regression

Excluded in the current review were studies examining pregnant or lactating women, children or adolescent populations, clinical populations (eg. people with inflammatory bowel diseases, diabetes, stroke, colorectal diseases, myocardial infarction, cancer), individual dietary components (eg. omega-3 or omega-6 fatty acids, alcohol) and animal or cell studies.

### Study selection and screening

The literature search was completed in May 2019 separately for each of the four databases and these were exported to Endnote X9. Duplicate articles were removed in Endnote by one author (MH). The remaining articles were then exported to RAYYAN where two researchers (MH and NB) reviewed the articles title, abstract and/or ‘full text’ for inclusion independently. Each researcher was blinded to the other researcher’s decisions. Once both reviewers completed screening, where there was a conflict in a decision, they met to reach an agreement on inclusion or exclusion of the article.

### Data extraction and quality assessment

The relevant data (where available) extracted for inclusion in the table were: author/s, year of publication, country, length of study for prospective studies, cohort name, study design, sample size, age of population, percentage of population that was female, dietary intake assessment method and dietary pattern assessment method, inflammatory marker/s assessed, confounders, statistical methodology and main results. The quality of studies was assessed using the National Institutes of Health Quality Assessment Tool for Observational Cohort and Cross-Sectional Studies [22]. This assessment tool was chosen as it has been widely used in systematic reviews [23–27]. The assessment tool has 14 assessment criteria. To score each study the criteria carried equal weighting resulting in a possible score from zero to 14 with higher scores representing higher quality studies. Data extraction and quality assessment was completed by one author (MH). Scores of < 7 were considered to have a high risk of bias, 7–10 a moderate risk of bias and 11–14 a low risk of bias. Details of the assessment criteria and results can be found in the Supplementary material.

In order to allow a summary description of the many dietary patterns using data-driven approaches, the three food groups with the highest contribution to each of the dietary pattern were extracted. This is a broad approach, however, allows for a description and comparison of the numerous data-driven dietary patterns. Based on these food groups the dietary pattern was assigned a category of Mediterranean if the top three food groups included components of the Mediterranean diet [28]; healthy if the top three food groups are listed as healthy according to the World Health Organisation’s Healthy Diet Fact sheet No. 394 [29].; unhealthy if the three food groups are not listed as healthy or mixed if the top three food groups are a mixture of Mediterranean, healthy and unhealthy foods. Studies utilising diet scores were also classified as Mediterranean if the diet score was based on a Mediterranean diet; national recommendations if the diet score was based on national recommendation; inflammatory index if the diet score was based on an inflammatory index; healthy if the diet score was based on any other diet score based on a known healthy dietary pattern; unhealthy if the diet score was based on dietary energy density and mixed if the diet score was based on the Inuit, Greenlandic or Palaeolithic diets. This allows a comparison between the ranges of dietary patterns identified in these studies.

## Results

**Fig. 1:**
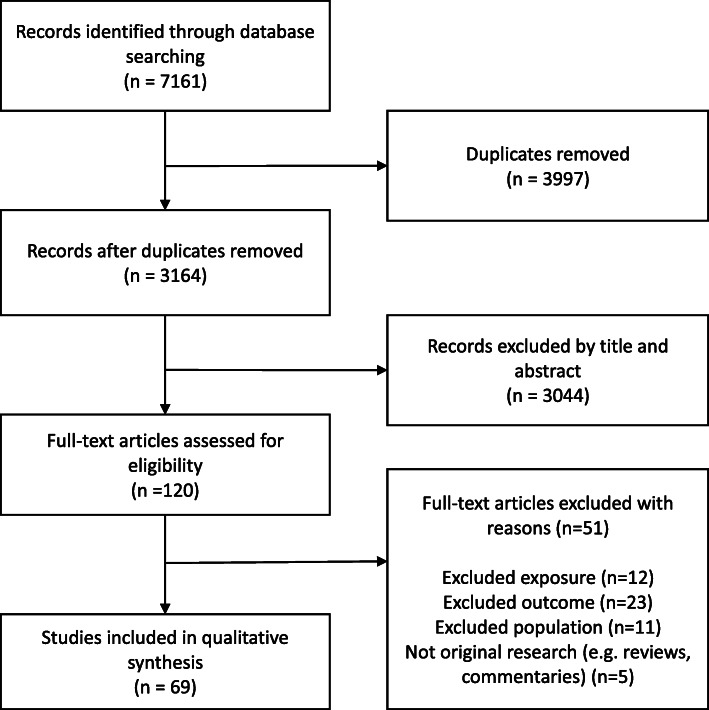
PRISMA flowchart outlining the search and screening process

The search of the databases identified 7161 studies (CINAHL Complete 1261, Embase 1899, MEDLINE Complete 2298, Global Health 1703) with 3164 studies remaining after duplicates were removed (Fig. 1). Sixty-nine studies met the inclusion criteria after screening (summarized in Supplementary Table 1) [17, 30–100].

The majority of studies identified were cross-sectional (*n* = 60) [17, 31–38, 40–42, 44–47, 49–53, 55–62, 64, 67–70, 72–75, 78–89, 91–100], followed by longitudinal studies (*n* = 9) [30, 39, 48, 63, 65, 66, 76, 77, 90]. The populations studied ranged in age from 18 years to 97 years with the majority (*n* = 59) examining populations 18 years and older. The remaining studies examined adults across mid and older age: there were 13 studies examining populations over age 50 years and of these, 5 examined populations over age 65 years. The majority of the studies were conducted in European countries (*n* = 26) [17, 30, 32, 33, 36, 40–42, 44, 45, 50, 57, 58, 60, 61, 65–67, 70, 77, 89, 91, 94, 96, 99, 100] and the United States America (USA) (*n* = 25) [31, 34, 38, 39, 46, 48, 49, 51–53, 62, 63, 68, 72, 75, 76, 82–85, 90, 92, 93, 97, 98]. There were 5 studies conducted in both Japan [37, 55, 69, 80, 81] and Iran [35, 47, 56, 64, 86], two in both Greenland [87, 88] and Korea [74, 79], and one each in Canada [73], China [95], Australia [59] and Taiwan [78]. The studies ranged in size from 120 participants in two cross-sectional studies of Italian men and women with a mean age of 60 years [82, 83] to 62,965 in a cross-sectional study of Taiwanese adult men and women > 40 years of age [78].

### Cross-sectional studies - scores

There were 39 cross-sectional studies that assessed dietary patterns using scores (Supplementary Table 1) [17, 31–33, 35, 36, 38, 41, 44, 45, 49–52, 56, 59, 61, 62, 67, 68, 70, 72, 78, 79, 82, 84–89, 91–94, 96–99]. There were 27 distinct scores utilized with 8 studies assessing multiple scores. Twenty studies assessed multiple inflammatory biomarkers. This resulted in 133 individual analyses being conducted across the 39 studies for associations between a diet score and a biomarker of inflammation.

Diet scores assessing the inflammatory potential of the diet were assessed in 9 studies that included 34 analyses [31, 79, 89, 92–94, 98, 99]. These scores included the DII by Shivappa et al. [90] in 18 analyses, the Empirical Dietary Inflammatory Index (EDII) by Tabung [92] in 12 analyses and the Adapted Dietary Inflammatory Index (ADII) by van Woudenbergh [94] in 4 analyses. Higher scores of these three indices indicate a more inflammatory diet. In 23 of the 34 analyses, higher inflammatory index scores were positively associated with higher inflammatory status and 11 reported no association. Examining individual biomarkers, in 7 of 12 analyses of CRP (across 9 studies), higher inflammatory diet scores were positively associated with CRP indicating more inflammation [32, 33, 79, 89, 92–94, 98, 99]. In 7 of 8 analyses of IL-6 (across 5 studies), higher inflammatory diet scores were positively associated with IL-6 indicating more inflammation [89, 92–94, 98]. In 5 of 7 analyses of TNF-α (across 4 studies), higher inflammatory diet scores were positively associated with TNF-α indicating more inflammation [92–94, 98]. In three of 5 analyses of adiponectin (across two studies), higher inflammatory diet scores were inversely associated with adiponectin indicating more inflammation [92, 93]. Fibrinogen was assessed in one analysis (in one study) and no association was reported between inflammatory diet scores and fibrinogen [89].

The Mediterranean diet was assessed in 14 studies including 31 analyses [17, 31, 33, 36, 38, 41, 44, 50, 52, 70, 85, 91, 96, 97] with the MedDietScore by Panagiotakos [17] being used in 17 analyses, the Mediterranean Diet Score (MDS) by Trichopoulou [18] being used in 10 analyses and the alternate Mediterranean Diet Score (aMED) [101] which is based on the MDS being utilized in 4 analyses. Twenty-one of the 31 analyses reported that higher adherence to a Mediterranean diet was inversely associated with inflammation and 10 reported no association. The most commonly assessed biomarker was CRP with 10 of 13 analyses reporting an inverse association between the Mediterranean diet and CRP indicating less inflammation [17, 33, 38, 41, 50, 52, 70, 85, 91, 96, 97]. In 3 of 6 analyses of TNF-α (across 5 studies), higher adherence to a Mediterranean diet was inversely associated with TNF-α indicating less inflammation [36, 41, 44, 50, 91]. In 3 of 4 analyses assessing IL-6 (across 4 studies), higher adherence to a Mediterranean diet was inversely associated with IL-6 indicating less inflammation [41, 44, 50, 52]. In 3 of 4 analyses assessing fibrinogen (across 3 studies), higher adherence to a Mediterranean diet was inversely associated with fibrinogen indicating less inflammation [17, 38, 41]. In 2 of 4 analyses assessing adiponectin (across 3 studies), higher adherence to a Mediterranean diet was positively associated with adiponectin indicating less inflammation [31, 50, 91].

The Healthy Eating Index (HEI) [102] and Alternative Healthy Eating Index (AHEI), a modified version of the HEI [103], were examined in 11 studies containing 20 analyses [31, 49, 51, 52, 56, 62, 68, 72, 84–86]. In 11 of 20 analyses higher adherence to the HEI or AHEI was inversely associated with an inflammatory biomarker and 9 found no association. In 9 of 13 analyses assessing CRP (across 10 studies), higher adherence to the HEI or AHEI was inversely associated with CRP indicating less inflammation [49, 51, 52, 56, 62, 68, 72, 84–86]. In 1 of 3 analyses assessing IL-6 (across 2 studies), higher adherence to the HEI was inversely associated with IL-6 indicating less inflammation [52, 62]. In 1 of 2 analyses assessing adiponectin (across 2 studies), higher adherence to the AHEI was positively associated with adiponectin indicating less inflammation [31, 62]. In 2 analyses (across 2 studies), assessing the association between adherence to the HEI and fibrinogen, no association was found [68, 86].

The remaining 50 analyses in 16 studies [31, 33, 35, 45, 52, 59, 61, 67, 68, 72, 78, 82, 87, 88, 92, 97] which examined 17 different diet scores, 25 found higher adherence to the assessed dietary pattern was positively associated with better inflammatory biomarker levels, 20 found no association and three were inversely associated with inflammatory biomarker status. Twenty-three analyses across 15 studies examined CRP [33, 35, 45, 52, 59, 61, 67, 68, 72, 78, 82, 87, 88, 92, 97] of which 12 reported improved biomarker levels, 9 reported no association and two reported poorer biomarker status. In 5 of 10 analyses assessing IL-6 (across 6 studies), higher adherence to the diet score was inversely associated with IL-6 [35, 52, 61, 67, 82, 92]. In 3 of 5 analyses assessing TNF-α (across 4 studies), higher adherence to the diet score was inversely associated with TNF-α [35, 45, 67, 92]. In 3 of 4 analyses assessing adiponectin (across 3 studies), higher adherence to the diet score was inversely associated with adiponectin [31, 67, 92]. In 2 of 4 analyses assessing fibrinogen (across two studies), higher adherence to the diet score was inversely associated with fibrinogen [68, 82].

Overall, in cross-sectional studies, diet scores assessing healthy diets or Mediterranean diets appear to be associated with inflammatory markers with 41 of 64 analyses reporting an inverse association between these diets and inflammatory biomarkers (Table 2). Inflammatory indices, measuring a pro-inflammatory diet, appear to be associated with inflammatory markers with 22 or 34 analyses reporting a positive association between an inflammatory diet and inflammatory biomarkers. Diet scores based on national dietary recommendations, such as the HEI, are less consistently associated with inflammatory biomarkers with 15 of 29 analyses reporting no association. Few analyses assessed unhealthy and mixed patterns.

**Table 2:** Summary of cross-sectional associations between diet score category and inflammatory markers in a general adult population

Diet score category	Inflammatory marker assessed	Inverse association *n*	No association *n*	Positive association *n*
**Inflammatory indices**^a^	Adiponectin		2	3
	CRP		5	7
	Fibrinogen		1	
	IL-6		1	7
	TNF-α		2	5
*Inflammatory indices – total*			**11**	**23**
Healthy	Adiponectin	2	2	1
	CRP	10	3	
	Fibrinogen	2	2	
	IL-6	3	3	
	TNF-α	2	1	
*Healthy totals*		**20**	**12**	**1**
Mediterranean	Adiponectin	2	2	
	CRP	10	3	
	Fibrinogen	3	1	
	IL-6	3	1	
	TNF-α	3	3	
*Mediterranean totals*		**21**	**10**	
National recommendation	Adiponectin	1		
	CRP	10	9	
	Fibrinogen		2	
	IL-6	2	4	
	TNF-α	1		
*National recommendation totals*		**14**	**15**	
Unhealthy	CRP			1
	IL-6			1
	TNF-α		1	
*Unhealthy total*		**0**	**1**	**2**
Mixed	CRP		1	2
*Mixed totals*			**1**	**2**

*CRP* C-reactive protein, *IL-6* Interleukin 6, *TNF-α* Tumor Necrosis Factor α

^a^ A higher score indicates a more inflammatory diet

### Cross-sectional studies – data-driven approaches

Twenty-three cross-sectional studies assessed dietary patterns using data-driven approaches. (Supplementary Table 1) [34, 37, 40, 42, 46, 47, 53, 55, 57, 58, 60, 64, 69, 72–75, 78, 80, 81, 83, 95, 100] There were 83 dietary patterns identified across the 23 studies with 59 unhealthy, 37 healthy, 33 mixed and 16 Mediterranean dietary pattern classifications. Eight of the 23 studies assessed multiple inflammatory biomarkers, resulting in 145 individual analyses being conducted across the 23 studies.

Twenty studies had unhealthy dietary patterns, which included 59 analyses [34, 37, 46, 47, 53, 55, 57, 58, 60, 64, 69, 72–75, 78, 80, 81, 83, 100]. In 10 of the 59 analyses higher adherence to an unhealthy dietary pattern was associated with poorer inflammatory biomarker status and in 3 of the 59 analyses was associated with better inflammatory biomarker status. No association was found between the unhealthy dietary patterns and inflammatory status in 46 of the 59 analyses. Fifteen studies had healthy dietary patterns, which included 37 analyses [34, 37, 42, 46, 47, 55, 60, 64, 69, 73, 74, 81, 83, 95, 100]. In 11 of the 37 analyses higher adherence to a healthy dietary pattern was associated with better inflammatory biomarker status and in one of the 37 analyses was associated with poorer inflammatory biomarker status. No association was seen between the healthy dietary pattern and inflammatory biomarkers in 25 of the 37 analyses. Fifteen studies had mixed dietary patterns, which included 33 analyses [37, 40, 46, 47, 55, 57, 60, 64, 69, 73, 74, 80, 83, 95, 100]. In one of the 33 analyses adherence to a mixed pattern was associated with better inflammatory biomarker status and in 4 was associated with poorer inflammatory biomarker status. No association was found between the mixed dietary pattern and inflammatory biomarkers in 28 of the 33 analyses. Eleven studies had a Mediterranean pattern, which included 16 analyses [37, 40, 42, 53, 57, 58, 72, 75, 78, 80, 100]. In 7 of the 16 analyses, adherence to a Mediterranean dietary pattern was associated with better inflammation biomarker status and in 9 analyses no association was reported. No analyses reported that the Mediterranean dietary pattern was associated with poorer inflammatory biomarker status.

In relation to specific biomarkers, CRP was assessed in 16 studies, which included 65 analyses [34, 40, 42, 46, 47, 53, 60, 72–75, 78, 80, 81, 83, 95]. In 6 of 65 analyses of CRP, higher adherence to a healthy pattern was inversely associated with CRP indicating less inflammation and in one analysis of 65 higher adherence to a healthy pattern was positively associated with CRP indicating more inflammation. In 8 of 65 analyses assessing CRP no association was reported. Eight analyses assessed the Mediterranean pattern and CPR. In 5 of 8 analyses, higher adherence to the Mediterranean pattern was inversely associated with CRP indicating less inflammation and three analyses found no association. No analyses found a positive association between the Mediterranean pattern and CRP. Twenty-nine analyses assessed an unhealthy pattern and CRP. In 5 of 29 analyses higher adherence to an unhealthy pattern was positively associated with CRP and in 3 of the 29 analyses higher adherence to an unhealthy pattern was inversely associated with CRP. In 21 of 29 analyses assessing an unhealthy pattern and CRP no association was reported. Thirteen analyses assessed a mixed pattern and CRP. In two of the 13 analyses higher adherence to a mixed pattern was positively associated with CRP indicating more inflammation and 11 analyses reported no association. No analyses reported an inverse association between the mixed pattern and CRP.

Adiponectin was assessed in 5 studies, which included 30 analyses [37, 55, 64, 69, 100]. In 2 of 12 analyses assessing a healthy dietary pattern, higher adherence to a healthy dietary pattern was positively associated with adiponectin, indicating less inflammation and 10 analyses reported no association. In 1 of 3 analyses assessing a Mediterranean dietary pattern, higher adherence to a Mediterranean dietary was positively associated with adiponectin and 2 analyses reported no association. In 1 of 7 analyses assessing an unhealthy dietary pattern, higher adherence to the unhealthy patterns was inversely associated with adiponectin indicating more inflammation and 6 analyses reported no association. In one of 8 analyses assessing a mixed dietary pattern, higher adherence to a mixed diet was positively associated with adiponectin and one was inversely associated with adiponectin. In 6 of 8 analyses no association was reported between a mixed dietary pattern and adiponectin.

IL-6 was assessed in 5 studies, which included 19 analyses [34, 47, 73, 75, 83]. In one of 4 analyses adherence to a healthy pattern was inversely associated with IL-6, while three analyses reported no association. The Mediterranean pattern was assessed in one analysis and no association was found with IL-6. In three of 9 analyses higher adherence to an unhealthy pattern was positively associated with IL-6, while 6 analyses found no association. In one of 5 analyses adherence to a mixed pattern was positively associated with IL-6, while 4 analyses found no association.

TNF-α was assessed in three studies, which included 13 analyses [34, 47, 73]. No association was reported between three healthy, 7 unhealthy or 3 mixed dietary pattern and TNF-α.

Fibrinogen was assessed in 5 studies, which included 14 analyses [42, 46, 53, 57, 58]. In one of two analyses a healthy pattern was inversely associated with fibrinogen indicating less inflammation and in one analysis no association was seen. In one of 4 analyses a Mediterranean pattern was inversely associated with fibrinogen and in three analyses no association was reported. No associations were reported between fibrinogen and the unhealthy pattern in 6 analyses and the mixed pattern in two analyses.

Overall, cross-sectionally, data-driven dietary patterns are less consistently associated with inflammatory markers compared to diet scores with 108 of the 145 analyses reporting no association (Table 3).

**Table 3:** Summary of cross-sectional associations between data-driven dietary pattern category and inflammatory markers in a general adult population

Data-driven category	Inflammatory marker assessed	Inverse association *n*	No association *n*	Positive association *n*
Unhealthy	Adiponectin		6	1
	CRP	3	21	5
	Fibrinogen		6	
	IL-6		6	3
	TNF-α		7	
*Unhealthy totals*		**3**	**46**	**10**
Healthy	Adiponectin	2	10	
	CRP	6	8	1
	Fibrinogen	1	1	
	IL-6	1	3	
	TNF-α		3	
*Healthy totals*		**11**	**25**	**1**
Mixed	Adiponectin	1	6	1
	CRP		11	2
	Fibrinogen		2	
	IL-6		4	1
	TNF-α		3	
*Mixed totals*		**1**	**28**	**4**
Mediterranean	Adiponectin	1	2	
	CRP	5	3	
	Fibrinogen	1	3	
	IL-6		1	
*Mediterranean totals*		**7**	**9**	

*CRP* C-reactive protein, *IL-6* Interleukin 6, *TNF-α* Tumor Necrosis Factor α

Unhealthy diet patterns were the most common diet classification with 46 of 59 analyses reporting no association between an unhealthy pattern and inflammatory markers. Healthy diet patterns were the next most common classification with 25 of 37 analyses reporting no association between a healthy diet and inflammatory markers. Mixed diet pattern classification reported no association in 28 of 33 analyses and a Mediterranean diet patterns in 9 of 16 analyses.

### Longitudinal studies – scores

There were 8 longitudinal studies assessing dietary patterns using diet scores, these ranged in duration from 1 to 15 years [30, 39, 48, 63, 65, 76, 77, 90]. Four of the studies assessed multiple diet scores [63, 65, 76, 77] and three assessed multiple inflammatory biomarkers [30, 48, 63]. This resulted in 34 separate analyses across the 8 studies. Analyses assessing higher adherence to the HEI and AHEI scores were conducted in 5 studies, which included 17 analyses [30, 48, 63, 76, 77]. In 6 of 17 analyses higher adherence to the HEI and AHEI were associated with improved inflammatory biomarker status and 11 found no change in studies ranging from two to 11.8 years. CRP was assessed in 5 studies, which included 9 analyses [30, 48, 63, 76, 77]. In 4 of 9 analyses higher adherence to the HEI and AHEI was associated with a decrease in CRP and 5 analyses reported no change in studies ranging from two to 11.8 years. Adiponectin was assessed in two studies, which included 5 analyses [48, 63]. In one of 5 analysis over 6 years, higher adherence to the AHEI was associated with an increase in adiponectin indicating an improvement in inflammatory biomarkers. In 4 analyses over 9.5 years higher adherence to the HEI or AHEI reported no change in adiponectin. IL-6 was assessed in two studies which included one analyses [30, 48]. In one of two analyses higher adherence to the AHEI was associated with an increase in adiponectin over 6 years and a further analysis over 6 years found no change in adiponectin. Adherence to the AHEI and TNF-α was assessed in one study with one analysis. Higher adherence to the AHEI saw no change in TNF-α over 6 years [48].

Adherence to the Dietary Approaches to Stop Hypertension (DASH) diet was assessed in three studies, which included 6 analyses [63, 76, 77]. In 4 of 6 analyses higher adherence to the DASH was associated with improved inflammatory biomarkers and two found no change. CRP was assessed in three studies, which included 4 analyses [63, 76, 77]. In two of 4 analyses higher adherence to the DASH was associated with a decrease in CRP, while two of 4 analyses reported no change in studies ranging from two to 11.8 years. Adiponectin was assessed in one study, which included two analyses [63]. In two of two analyses adherence to the DASH was associated with an increase in adiponectin indicating an improvement in inflammatory biomarkers over 9.5 years.

The inflammatory potential of the diet was assessed in three studies, which included 4 analyses [39, 65, 90], two using the DII, one using the ADII and one using an Inflammatory Index (II) developed by Cavicchia et al. [39]. In one of 4 analyses a more inflammatory diet was associated with an increase in CRP over 1 year [90]. No change was seen in the other three analyses ranging from one to 12 years.

A Mediterranean diet was assessed in two studies that included 5 analyses [63, 76]. The aMED was assessed 4 times in one study over 9.5 years [63] and the MDS once in another study over 2 years [76]. CRP was assessed in two studies, which included three analyses [63, 76]. In three of 5 analyses higher adherence to a Mediterranean diet was associated with a decrease in CRP in in studies of over 2 or 9.5 years [63, 76]. Adiponectin was assessed in one study, which included two analyses [63]. In two (of two) analyses higher adherence to the aMED was not associated with a change in adiponectin over 9.5 years. The Healthy Diet Indicator and the American Heart Association Diet Score were each assessed in one study with each study including one analyses for association with CRP [76, 77]. No changes in CRP were found.

Overall, longitudinal studies assessing diet scores were less consistently associated with inflammatory markers than cross-sectional studies with 20 of the 34 studies reporting null findings (Table 4). Diet scores based on national recommendations were most commonly assessed and in 12 of 18 analyses adherence to a diet based on national recommendations reported no change in inflammatory status. In 4 of 7 analyses adherence to a healthy diet scores and in 3 of 5 analyses adherence to a Mediterranean diet was associated with improvements in inflammatory markers. In 3 of 4 analyses an inflammatory diet was not associated with a change in inflammatory status.

**Table 4:** Summary of longitudinal associations between diet score category and inflammatory markers in a general adult population

Diet score category	Inflammatory marker assessed	Improved inflammatory status *n*	No change *n*	Reduced inflammatory status *n*
National recommendations	Adiponectin	1	4	
	CRP	4	6	
	IL-6	1	1	
	TNF-α		1	
*National recommendation totals*		**6**	**12**	
Healthy	Adiponectin	2		
	CRP	2	3	
*Healthy totals*		**4**	**3**	
Mediterranean	Adiponectin		2	
	CRP	3		
*Mediterranean totals*		**3**	**2**	
Inflammatory indices	CRP		3	1
*Inflammatory indices totals*			**3**	**1**

*CRP* C-reactive protein, *IL-6* Interleukin 6, *TNF-α* Tumor Necrosis Factor α

### Longitudinal studies – data-driven approaches

One study was identified that examined associations between data-driven dietary patterns and CRP over 12 years [66]. A Mediterranean pattern was associated with a decrease in CRP over 12 years. Three mixed patterns were also identified where one (containing eggs, poultry and processed meats) was found to be associated with an increase in CRP while the remaining two (one containing fruit, fruit juices and seafood the other containing seafood, processed meats and organ meats) found no associations [66].

### Quality of studies

Overall, 7 (10%) studies included in this review had a low risk of bias, 51 (74%) had a moderate risk of bias and 11 (15%) had a high risk of bias. (Supplementary Table 1) The 11 studies with a high risk of bias were all cross-sectional studies, while the 7 low-risk studies were longitudinal. The objective nature of the measurement of the inflammatory marker outcomes helped to reduce the risk of bias in these studies with all 69 (100%) observational studies reporting well. The self-reported nature of the dietary exposure in the observational studies introduces misreporting bias of dietary intake in these studies with all 69 (100%) relying on self-reported dietary data [104]. Most observational studies (88%) adjusted for appropriate confounding variables. Loss to follow-up was poorly reported with only two of the 9 (22%) longitudinal studies reporting this clearly. The participation rate was also poorly reported with 14 (20%) of the observational studies clearly stating this.

The objective nature of the inflammatory outcome measures helped reduce the risk of bias in these studies.

Cross-sectional studies with a high risk of bias that examined diet scores had a larger proportion reporting null findings (57% of analyses) compared to studies with low/medium risk of bias (35% of analyses). Cross-sectional studies with a high risk of bias that examined data-driven dietary patterns did not have such a difference between studies with similar proportion reporting null findings across studies with high risk of bias (71% of analyses) and low/medium risk of bias (75% of analyses).

## Discussion

The aim of this review was to summarise the current evidence from observational studies examining associations between dietary patterns and inflammatory biomarkers in the general adult population. Overall, this review found strong evidence for adherence to a healthy, Mediterranean and anti-inflammatory diet and lower levels of inflammation. However, studies using data-driven dietary patterns are less consistently associated with inflammatory biomarkers. Additionally, there is strongest evidence for an association between dietary patterns and inflammation in cross-sectional studies, with a smaller number of longitudinal studies reporting less consistent results. Cross-sectionally and longitudinally, data-driven dietary patterns are less consistently associated with inflammatory markers compared to pre-defined diet scores.

This review found that a healthy, Mediterranean and anti-inflammatory diet is associated with lower inflammation which is consistent with the review by Barbaresko et al. in 2013 [20] which concluded that cross-sectionally there was some evidence for the association between dietary patterns and inflammatory biomarkers. The additional cross-sectional studies identified for this review strengthen and support their conclusions. This review also supports the conclusion of the meta-analysis by Schwingshackl et al. in 2014 [19], which concluded the Mediterranean diet is anti-inflammatory. This adds to the body of literature showing specific dietary patterns, nutrients and foods may reduce inflammation and possibly protect from inflammatory diseases. The previous review was of cross-sectional studies, and looked at both clinical and other populations [20]. The current review focussed on a general adult population and included longitudinal studies to investigate the influence of diet on inflammation.

Although there were a wide variety of methodologies and scores used to assess dietary patterns included in the current review, diet scores assessing adherence to inflammatory indices, healthy or Mediterranean diets were most consistently associated with inflammatory status. It should be noted that these were also the most commonly assessed scores in the literature, which may explain the high number of positive associations reported between these types of measures and inflammatory biomarkers. However, there is qualitative overlap between these three scores that should also be considered. For example, the anti-inflammatory components of the inflammatory indices [16] include vitamins, minerals and fatty acid which are consumed as foods (including vegetables, fruits and seafood) that are components of Mediterranean [17, 18] and the healthy diet scores. There are substantial correlations in what these diet scores are measuring, albeit by different methodologies, so some consistency in findings across these scores should be expected, as observed in this review.

There were significant differences identified between the frequency with which diet scores and data-driven approaches were associated with inflammatory markers, with 42% of analyses using diet score reporting null findings compared with 74% of data-driven pattern analyses. The difference seen may be due to the different approaches these two methodologies employ. Diet scores are less subjective as they are pre-defined and often based on known healthy diets such as the Mediterranean diet or diets that are known to prevent diseases. In contrast, data-driven approaches look for patterns in the data and are more likely to describe what people are eating rather than measure compliance to a healthy dietary pattern. The authors of the current review acknowledge the approach taken to classify the data-driven dietary patterns using three food groups, while allowing comparison of the many data-driven patterns, may reduce the nuance between the patterns. An evaluation of these two different methodologies by Ocké M. noted that the objective nature of diet scores are useful to assess adherence to dietary guidelines and known healthy diets, while data-driven approaches are appropriate for understanding patterns within a population but may not be as effective in predicting a health outcome [105]. The results of this review support this observation in that diet scores show a more consistent association with inflammatory biomarkers than data-driven approaches.

Cross-sectional studies examining diet scores with a high risk of bias were more likely to report no associations between diet and an inflammatory biomarker compared to data-driven approaches. Therefore, if considering only low/medium risk of bias studies there is larger divergence in the frequency with which diet scores and data-driven approaches were associated with inflammatory markers.

Results appear to vary by study design, where diet scores were more consistently associated with better inflammatory status in cross-sectional studies compared to longitudinal studies. This may be due to the short-term nature of the inflammatory response. For example, the most frequently measured inflammatory markers in this review were CRP and IL-6, with half-lives of 62 and 15 h respectively [106]. However, if an individual’s habitual diet is unlikely to vary substantially over time [107] you would expect cross-sectional associations to be mirrored longitudinally. This is not what was observed in this review and may be because the associations seen cross-sectionally are not causal or may be due to some short-term variability in dietary patterns rather than longer term changes in habitual diets.

Overall this review finds that the dietary pattern method chosen in the studies is a significant factor in determining if associations are seen between a dietary pattern and inflammation. Healthy, Mediterranean and anti-inflammatory diets are more frequently associated with lower levels of inflammation compared to other diet scores and data-driven dietary patterns. Hypothesis or literature driven diet scores are more consistently associated with inflammation and may be a more appropriate dietary pattern methodology when assessing health outcomes. While data-driven approaches may identify patterns of dietary behavior within a population, this methodology may not always identify patterns associated with health outcomes. Additionally, the majority of the observational studies identified for this review involved the secondary analysis of existing data, and the prime purpose of these studies was not to examine the association between dietary patterns and inflammation.

CRP was the most frequently examined inflammatory biomarker. The majority of analyses reported an association between a healthier diet assessed by various dietary patterns or diet scores and lower CRP, indicating less inflammation. IL-6 followed a similar pattern to CRP, however, associations between dietary patterns and IL-6 were less consistent. This inconsistency may be due to the shorter half-life of IL-6 such that CRP is likely to remain in the serum longer and because IL-6 triggers production of CRP by the liver [106]. This highlights the complex nature of inflammatory pathways when trying to assess overall inflammatory status. In this review less than half the studies examined more than one inflammatory biomarker. In order to improve the quality of these studies and better understand overall inflammatory status, studies examining a range of inflammation biomarkers would provide a more comprehensive insight into the effect of diet on inflammation.

The strengths of this review include its comprehensive focus incorporating observational study designs in a general adult population. The review includes a comprehensive assessment of dietary pattern methodologies, including both diet scores and data-driven approaches. This review summarizes new evidence that has been published since previous reviews [19, 20] and includes longitudinal studies that were not previously available. The objective measurement of the biomarker outcomes strengthens the findings in this review. The large number of cross-sectional studies and the limited number of longitudinal studies limit the strength of the conclusions drawn as does the heterogeneity of the exposures and outcomes which meant a meta-analysis could not be conducted to estimate an effect size. The self-reported nature of the dietary exposure in the observational studies is a limitation due to known misreporting bias of dietary intake [104].

This review identified a number of research gaps. Studies frequently assessed only one inflammatory biomarker at one time-point and future research should focus on examining multiple inflammatory makers concurrently with repeat measures to better understand the complex biological inflammatory mechanisms. Given the studies identified for this review were predominantly cross-sectional, future research should focus on longitudinal designs using a potential outcomes approach [108] using appropriate existing datasets. This could permit a causal effect to be inferred for associations between diet and inflammation.

## Conclusions

Adherence to healthy dietary patterns, including the Mediterranean and the inflammatory diet are cross-sectionally associated with lower levels of inflammation however these findings were not replicated in longitudinal studies. Further work is required to establish a coherent understanding of the impact of diet on the numerous inflammatory markers and the timeframe over which diet may impact inflammatory status. Dietary patterns using diet scores, based on known healthy diets, are more likely to be associated with less inflammation compared to patterns derived using data-driven methods which are more likely to explain variation within the data than predict health outcomes. The evidence base for a causal effect of dietary patterns on inflammation would be strengthened by using a potential outcomes approach in long-term longitudinal studies with repeat measures of both diet and inflammation.

## Supplementary Information

**Additional file 1: Supplementary Table 1**. Summary of studies examining dietary patterns and inflammatory biomarkers.

## Data Availability

All data generated or analysed during this study are included in this published article (and its supplementary information files).

## Abbreviations

ADII: Adapted dietary Inflammatory index
AHEI: Alternative Healthy Eating Index
aMED: alternate Mediterranean Diet Score
CRP: C-reactive protein
CVD: Cardiovascular disease
DII: Dietary Inflammatory Index
EDII: Empirical dietary inflammatory index
FA: Factor analysis
FFQ: Food frequency questionnaire
HEI: Healthy Eating Index
IL-6: Interleukin 6
PCA: Principal component analysis
PRISMA: Preferred reporting items for systematic reviews and meta-analyses
RCT: Randomized controlled trial
RRR: Reduced rank regression
T2D: Type-2 diabetes
TNF-α: Turmour necrosis factor alpha
USA: United States of America
