# Supplementary material for: Dietary patterns and associations with biomarkers of inflammation in adults: a systematic review of observational studies
Source: Nutr J. 2021 Mar 12;20:24. doi: 10.1186/s12937-021-00674-9 (PMC7955619; doi:10.1186/s12937-021-00674-9)
Supplement: Supplementary file 1 — Additional file 1: Supplementary Table 1. Summary of studies examining dietary patterns and inflammatory biomarkers. [file 12937_2021_674_MOESM1_ESM.docx]

**Supplementary table 1: Summary of studies examining dietary patterns and inflammatory biomarkers**

| *Cross-sectional studies* | | | | | | | |
| --- | --- | --- | --- | --- | --- | --- | --- |
| Authors, Year, Country, Cohort, Reference | **Sample size, (% women),**  **Age** | **Diet collection method,**  **Diet assessment** | **Outcome variable/s** | **Confounders examined** | **Statistical methodology** | **Results, Odds ratio or β coefficient, 95% confidence intervals, p-values** | **Quality score** |
| AlEssa, H et al 2017 USA Women’s Lifestyle Validation Study  (31) | 775 (100%) mean ~ 64 yrs | 152-item SFFQ DASH, aMED, aHEI | Adiponectin | A, AFB, ALC, DBM, DIAB, ETH, FHD, FHM, HRT, MPS, PA, SM, STA, VIT | Geometric means from multiple linear regression models for quartiles of diet scores and adiponectin | No associations found | 7 |
| Alkerwi, A et al 2014 Luxembourg ORISCAV-LUX  (32) | 1352 (52%) 18-69 yrs | 134 item FFQ  DII | CRP | A, EDU, PA, SES, SM, SX | Multivariate linear regression | No associations found | 7 |
| Alkerwi, A et al  2015  Luxembourg  ORISCAV-LUX  (110) | 1,352 (52%)  18-69 yrs | 134 item FFQ  RCI, DQI-I, DASH, MDS, DII | CRP | A, EDU, EI, PA, SM, SX | GLM dietary patterns and CRP | No associations found. | 8 |
| Anderson, AL et al  2012  USA  The Health, Aging and Body Composition (Health ABC) Study  (34) | 1751 (53%) 70-79 yrs | 108 item FFQ CA-6 (healthy foods, breakfast cereal, meat & alcohol, sweets & desserts, refined grains, high-fat dairy) | CRP  IL-6 TNF-α | A, BMI, CS, EDU, EI, ETH, PA, SM, SX | Multiple linear regression to compare outcome means to the ‘health foods’ cluster and biomarkers | The ‘healthy foods’ cluster had significantly lower interleukin-6 compared to ‘sweets and desserts’ and ‘high-fat dairy products’. | 8 |
| Azadbakht L et al. 2016 Iran (35) | 1,036 (100%) >30 yrs | 106 item FFQ  DED | CRP  IL-6 TNF-α | A, BMI, DIA, HRT, MPS, PA, SES, SM | ANCOVA for DED quartiles and biomarkers | Highest quartile of DED was associated with increased CRP (p = 0.04), TNF-α (p = 0.03) and IL-6 (p < 0.01). | 8 |
| Azzini, E et al 2011 Italy (36) | 164 (67%) 20-40 yrs | 4-day diary  MDS | TNF-α | - | ANOVA for tertiles of MDS and TNF-α | Highest tertile of MDS was associated with decreased TNF-α (p < 0.05). | 7 |
| Bahari, T et al 2017 Japan Japan Multi-Institutional Collaborative Cohort (J-MICC) Study (37) | 697 35-69 yrs | FFQ  PCA-5 (Vegetable, High-fat, Seafood, Bread & dairy, Protein) | Adiponectin | A, ALC, BMI, EI, MPS, PA, SM | Path analysis for each diet pattern and adiponectin by sex | In women, higher adherence to the ‘bread & dairy’ pattern was associated with higher adiponectin (p=0.015). | 8 |
| Carter, S et al 2010 USA  Third National Health and Nutrition Examination Survey  (38) | 13,197 ( 18-90 yrs | NHANES III FFQ + 24-hour dietary recall  MedDietScore | CRP Fibrinogen | A, BMI, ETH, PA, SM, STA | Multiple linear regression | In men >45 yrs higher MedDietScore was associated with lower CRP (p=0.0034) and fibrinogen (p=0.0028). | 8 |
| Centritto, F et al 2009 Italy  The Moli-sani Project (40) | 7243 Adults >35 yrs | EPIC FFQ PCA-(Olive Oil & Vegetables, Pasta & Meat, Eggs & Sweets) | CRP | A, BMI, EI, PA, SES, SM, SX, TRI | Multiple linear regression | Higher adherence to the ‘Olive Oil & Vegetables’ pattern was associated with lower CRP (p=0.018), Higher adherence to the ‘Pasta & Meat’ (p<0.0001) and ‘Eggs & Sweets’ (p<0.0001) patterns was associated with higher CRP | 9 |
| Chrysohoou, C et al 2004 Greece The ATTICA study (111) | 3,042  (50%) 18-87 yrs | FFQ  MedDietScore | CRP IL-6 Fibrinogen TNF-α | A, BMI, CHD, DIA, EDU, HC, HYP, PA, SM, SX | Multiple linear regression | Highest tertile of MedDietScore was associated with lower CRP (20% lower, p = 0.015), IL-6 (17% lower, p = 0.025) and fibrinogen (6% lower, p = 0.025) | 9 |
| Corley, J. et al 2015  UK Lothian Birth Cohort 1936 (42) | 792  (52%) 70 yrs | 168 item FFQ  PCA- (Mediterranean; Health-aware) | CRP Fibrinogen | A, BMI, CVD, HDL, IQ, PA, SES, SM, SX | Logistic regression for diet and CPR Linear regression for diet and fibrinogen | Higher adherence to the Health-aware pattern was associated with lower CRP, (OR = 0·82, (0·68, 0·99)).  Higher adherence to the Mediterranean pattern was associated with lower fibrinogen. (β = -0.100, p < 0.05) | 8 |
| Dedoussis, GV et al 2008 France, Greece, Germany, Italy Poland ZINCAGE population study (44) | 957 ( %) >60 yrs | FFQ MedDietScore | IL-6 TNF-α | A, CON, SM | Multiple linear regression | No associations found. | 6 |
| Dias, JA et al 2015 Sweden The Malmo Diet and Cancer (MDC) cohort (45) | 667 (59%) 63-68 yrs | 7-day menu book & 168-item quantitative diet questionnaire DQI-SNR | CRP TNF-α | A, EI, PA, SEA, SM, SX, WS | GLM | Higher adherence to the DQI-SNR was associated with lower CRP (p<0.05) and lower TNF-α (p<0.05) | 8 |
| Eilat-Adar, S et al 2009 USA Genetics of Coronary Artery Disease in Alaska Natives (GOCADAN) (46) | 1214 (56%) mean 41.7 yrs | 97 item FFQ PCA-( traditional, Western, purchased healthy, beverages & sweets) | CRP Fibrinogen | A, ALC, BMI, CAN, CHD, EDU, EI, HYP, PA, SM, STA, SX | Multiple linear regression | Higher adherence to the ‘beverages & sweets’ pattern was associated with lower CRP (p=0.04). Higher adherence to the ‘purchased healthy foods’ pattern was associated with lower Fibrinogen (p=0.00). | 8 |
| Esmaillzadeh, A et al 2007 Iran  (47) | 486 (100%) 40-60 yrs | 168 item SFFQ PCA-(healthy, western, traditional) | CRP TNF- α IL-6 | A, BMI, DIA, EI, HRT, MPS, PA, SM, WC | Multiple linear regression | Higher adherence to the ‘healthy’ pattern was associated with lower CRP (β = -0.05, p=0.011). Higher adherence to the ‘western’ pattern was associated with higher CRP (β = 0.07, p<0.001). Higher adherence to the ‘traditional’ pattern was associated with higher IL-6 (β = 0.04, p=0.045). | 9 |
| Ford, ES et al 2005  USA Third National Health and Nutrition Examination Survey  (49) | 13,811 (~51%) >20 yrs | 24-hr recall HEI | CRP | A, ASP, BMI, DIAB, EDU, ETH, EI, PA, SM, WHR | Proportions and means across quintiles of HEI scores and CRP | In women higher HEI scores were associated with lower CRP (p=0.005) | 8 |
| Fragopoulou, E et al 2010  Greece  The ATTICA study (50) | 532  (42%) mean 41 yrs | EPIC Greek FFQ  MedDietScore | CRP IL-6 TNF-α Adiponectin | A, BMI, PA, SM, SX | ANOVA tested for variance in outcomes between MDS tertiles | Highest MedDietScore tertile was associated with lower CRP (2.0 ±2.7, 1.4 ±2.1, p < 0.01) lower IL-6 (1.6 ±0.6, 1.2 ±0.4, p < 0.001) lower TNF-α (7.8 ±4.8, 4.6 ±4.6, p < 0.001) and higher adiponectin (3.4 ±1.9, 4.8 ±2.0, p < 0.001) | 9 |
| Frazier-Wood, A et al 2015  USA The National Health and Nutrition Examination Survey  (51) | 9797 (46%) >20 yrs | 1 x 24-hr recall HEI | CRP | A, ALC, EDU, ETH, INC, MPS, PA, SM | Population ratio approach | No association found. | 7 |
| Fung, TT et al 2005  USA  Nurses’ Health Study (52) | 690 (100%)  43-69 yrs | 140 item SFFQ HEI  AHEI DQI-R  RFS aMED | CRP IL-6 | A, ALC, BMI, EI, PA, SM | Multivariate linear regression | Higher AHEI scores were associated with lower CRP (β = -0.36, p<0.01) and lower IL-6 (β = -0.30, p<0.01).  Higher aMED scores were associated with lower CRP (β = -0.36, p<0.01) and lower IL-6 (β = -0.29, p<0.01). | 7 |
| Fung, TT et al 2001  USA Health Professionals Follow-up Study  (53) | 466 (0%) 40-75 yrs | 130 item SFFQ  FA-(Western, Prudent) | CRP Fibrinogen | A, ALC, EI, PA, SM, TV | Pearson partial correlation coefficients | Higher adherence to the Western dietary pattern was associated with higher CRP (0.22, p<0.0001) | 7 |
| Guo, H et al  2012 Japan (112) | 702  (0%) mean 45 yrs | 75 item FFQ  PCA- (Japanese; Sweets/fruits; Izakaya | Adiponectin | A, BMI, DPFS, DS, EDU, EI, GLU, HDL, OCC, PA, SBP, SM, SP, SX, TC, TRI | ANCOVA for dietary pattern tertiles and adiponectin | Highest tertile of the Japanese pattern was associated with higher adiponectin (5.24, (4.84, 5.69), 5.95, (5.47, 6.46) p<0.001) and Izakaya pattern with lower adiponectin (6.02, (5.56, 6.51), 5.47, (5.05, 5.92), p=0.03) | 9 |
| Haghighatdoost, F et al 2013  Iran Isfahan Healthy  Heart program (IHHP)  (56) | 9568 (50%)  Mean 39 yrs | 48 item FFQ  HEI | CRP | - | Linear regression | Higher HEI score was associated with lower CRP in men (β= -0.07, p<0.001) and women (β= -0.07, p<0.001). | 8 |
| Hamer, M et al  2010  United Kingdom (113) | 2,931 (65%) mean 49 yrs  (Low income population) | 3 x 24hr recall  FA (  fast food; health aware; traditional; sweet) | CRP | A, BMI, EDU, MRB, ODP, PA, SM, SX | GLM | Higher adherence to the ‘health aware’ pattern was associated with lower CRP levels (β = -0.05, (-0.10, 0.00), p=0.023) | 9 |
| Heidemann, C et al 2011 Germany  German Health Interview and Examination Survey (subset) (58) | 4025  18-79 years | Dietary Interview Software for Health Examination Studies (DISHES 98) face to face interview.  PCA-(Processed foods, Health-conscious) | Fibrinogen | A, EI, PA, SES, SM, SX | Multiple linear regression | No association found. | 9 |
| Hickling, S et al  2008  Australia  Busselton Health Study  (59) | 4077 (56%)  17-97 years | Short dietary questions CDS | CRP | A, ALC, BMI, HRT, OCT, PA, SM | Multiple linear regression | Higher CDS was associated with lower CRP in men (β = -0.093, p<0.0001) and women ( β = -0.056 p = 0.0003) | 7 |
| Hlebowicz, J et al  2011  Sweden  Malmo Diet and Cancer study  (60) | 4999 (59%)  45-68 yrs | Diet history interview  CA-( Many foods & drinks, Fibre-rich bread, Low-fat & high-fibre foods, White bread, Milk fat, Sweets & cakes) | CRP | A EI, PBF, SEA, WHR | Multiple linear regression | No associations found. | 8 |
| Hoebeeck, LI et al 2011  Belgium The Asklepios Study  (61) | 2487 (51.7%)  35-55 yrs | SFFQ Flemish FBDG | CRP IL-6 | A | Spearman correlation for FBDG and CRP.  GLM for FBDG and IL-6. | Higher FBDS was associated with lower IL-6 in men (β = -2.8 (-4.6, -1.0), p < 0.01) | 7 |
| Huang, T et al  2016 USA Nurses’ Health Study (114) | 831 (100%) mean 45 yrs | SFFQ  AHEI | CRP IL-6 Adiponectin | A, BMI, EI, HRT, MPS, PA, SM | Multivariable-adjusted least-squares geometric means for AHEI quintiles and biomarkers | Highest AHEI quintile was associated with higher adiponectin, (p = 0.002) | 8 |
| Jafari-Vayghan, H et al  2015  Iran  (64) | 150 (50%) 25-50 yrs | 132 item SFFQ  FA-(Western, Healthy, Mixed, Traditional) | Adiponectin | A, BMI, SX, WC | Multivariate linear regression | Higher adherence to the Western pattern was associated with lower adiponectin (β = -1.43 (±1.10), p=0.02) | 6 |
| Kanerva, N et al  2014 Finland  DILGOM and the Helsinki Birth Cohort Study (HBCS)  (67) | DILGOM-4,579  HBCS-1911  25-74 yrs | FFQ  BSDS | CRP  IL-6  Adiponectin  TNF- α | A, DBM, EI, EDU, OBM, PA, SM, STA, SX, WC | Multivariate linear regression | DILGOM: Higher BSDS was associated with lower adiponectin (p=0.006) and lower CRP (p=0.006).  HBCS: Higher BSDS was associated with lower CRP (p=0.004). | 9 |
| Kant, AK et al  2005  USA  National Health and Nutrition Examination Survey III  (68) | 8719 (50%)  ≥20 yrs | 24-hr recall  HEI, RFS, DDS-R | CRP  Fibrinogen | A, ALC, BMI, EDU, ETH, PA, SM | Multivariate linear regression | Higher RFS scores were associated with lower fibrinogen (β = -0.009 (±0.007), p=0.02).  Higher HEI (β = -0.001 (±0.000), p=0.04), RFS (β = -0.007 (±0.003), p=0.02) and DDS-R (β = -0.018 (± 0.008), p=0.04) scores were associated with lower CRP. | 7 |
| Kashino, I et al  2015  Japan (115) | 509  (42%) 20-65 yrs | BDHQ  PCA- (Japanese; Westernized Breakfast; Meat) | Adiponectin | A, BMI, CHT, DOM, EI, PA, SM, SX | Multiple linear regression | No associations found | 9 |
| Kelaiditi, E et al  2016  UK  (70) | 1658 (100%)  18-79 years | 131-item FFQ  MDS | CRP | A, AID, BMI, EI, HRT, MIS, PA, PRO, SM | Multivariate linear regression | No associations found | 6 |
| Ko, BJ et al  2016  USA (116) | 166  (51%) mean 46 yrs | 110 item FFQ  AHEI, DASH  PCA- (Western; Prudent) | CRP | A, ALC, BMI, EDU, EI, ETH, INC, MS, PA, SM, SX | Multiple linear regression | Higher AHEI (β = -0.21, p=0.018) and DASH (β = -0.20, p=0.023) scores were associated with lower CRP. A “Western” dietary pattern was associated with higher CRP (β = 0.041, p=0.023) | 6 |
| Labonté, M et al  2014  Canada Nunavik Inuit Health Survey  (73) | 666 (55%)  Mean 36.4 yrs | FFQ  PCA-(Traditional, Western, Nutrient-poor food, Healthy) | CRP  IL-6  TNF- α | CRP: A (nutrient-poor A only)  IL-6: A, SX, WC  TNF- α: A, SX (Traditional and nutrient-poor A only) | Multivariate linear regression | Higher adherence to the Nutrient-poor pattern was associated with lower CRP (p<0.0001). | 7 |
| Lee, Y et al  2014  Korea  (74) | 7574 (52%)  Mean 51.1 yrs | 103-item FFQ  FA-(Fruit, Vegetable, Meat, Coffee) | CRP | A, ALC, EDU, SM, SX, WC | Linear trend test | Higher adherence to vegetable pattern was associated with lower CRP (p=0.01) | 7 |
| Lopez-Garcia, E et al  2004  USA  Nurses’ Health Study I cohort  (75) | 732 (100%)  43-69 yrs | SFFQ  FA-(Prudent, Western) | CRP  IL-6 | A, ALC, BMI, PA, SM | Multivariate linear regression | Higher adherence to the Western pattern was associated with higher CRP (β = 0.10, p=0.02).  Higher adherence to the Prudent pattern was associated with lower CRP (β =  -0.10, p=0.02) | 6 |
| Muga, M et al  2016  Taiwan (78) | 62,965  (48%) >40 yrs | SFFQ  TDP  PCA- (vegetable / fruit, meat / processed) | CRP | A, ALC, BMI, CVD, DBP, EDU, MS, PA, SBP, SM, SX, WC | Multivariable linear regression | Highest quintile of the TDP was associated with lower CRP (β = -0.017. (-0.03, -0.004), p<0.01)  Higher vegetable/fruit dietary pattern associated with lower CRP, (β = -0.030 (-0.043, -0.017), p<0.001) | 8 |
| Na, W et al  2018  Korea  Korean Genome and Epidemiology Study (KOGES)  (79) | 28,086 (70%)  40-79 yrs | 2 x 24-hr recall DII | CRP | A, BMI, BP, EDU, EI, OCT, PA, SM, SX | Logistic regression for DII and CRP ≤3mg/L and >3mg/L | Highest quartile of DII score was associated with higher odds of elevate CRP (OR 1.241 (1.071, 1.438), p=0.002) | 6 |
| Nanri, A et al  2008  Japan  (80) | 7,802 (58%)  50-74 yrs | 60 item FFQ  PCA-(Healthy, High-fat, Seafood, Westernised breakfast) | CRP | A, ALC, BMI, PA, SM | Multivariate linear regression | Higher adherence to the healthy pattern was associated with lower CRP in men (p=0.0006) and women (p=0.0004). | 8 |
| Nanri, A et al  2011  Japan  Japan Multi-Institutional Collaborative Cohort Study (J-MICC Study)  (81) | 9545 (59%)  40-69 yrs | 47 item FFQ  FA-(Healthy, Western, Seafood, Bread, Dessert) | CRP | A, ALC, SM, PA, BMI | Multivariate linear regression | Higher adherence to the healthy (p=0.01) and dessert (p<0.01) patterns was associated with lower CRP in men. Higher adherence to the seafood pattern was associated with higher CRP in men (p=0.02). | 8 |
| Nettleton, J et al  2006  USA  Multi-Ethnic Study of Atherosclerosis (MESA)  (83) | 5089 (53%)  45-84 yrs | 120 item FFQ  FA-(fats & processed meats, vegetables & fish, beans, tomatoes & refined grains, whole grains & fruit) | CRP  IL-6 | A, EDU, EI, ETH, PA, SC, SM, SX, VIT, WC | Multivariate linear regression | Higher adherence to the fats & processed meats pattern was associated with higher CRP (β = 0.048 ± 0.023, p<0.05) and higher IL-6 (β = 0.025 ± 0.014, p<0.05).  Higher adherence to the whole grains & fruit pattern was associated with lower CRP (β = -0.060 ± 0.018, p<0.05) | 9 |
| Nettleton, J et al  2008  USA  Multi-Ethnic Study of Atherosclerosis (MESA)  (82) | 5089 (53%)  45-84 yrs | 120 item FFQ  CHDP, SHDP | CRP  IL-6  Fibrinogen | A, EDU, EI, ETH, PA, SC, SM, SX, VIT, WC | Multivariate linear regression | Higher adherence to the CHDP was associated with lower CRP (β = -0.07 ± 0.02, p<0.05), lower IL-6 (β = -0.05 ± 0.01, p<0.05) and lower fibrinogen (β = -0.01 ± 0.003, p<0.05). | 8 |
| Nicklas, TA et al  2012  USA  National Health and Nutrition Examination Survey 2001-2008 (NHANES)  (84) | 18,988 (49%)  ≥19 yrs | 24-hr dietary recall  HEI | CRP | A, ALC, BMI, ETH, EI, INC, PA, SM, SX | Multivariate linear regression | Higher HEI scores were associated with lower CRP (p=0.002) | 7 |
| Panagiotakos, DB et al  2006  Greece  The ATTICA Study  (17) | 3,042 (50%)  ≥18 yrs | SFFQ  MedDietScore | CRP  Fibrinogen | A, EDU, EI, PA, SM, SX | Multivariate linear regression | Higher MedDietScore was associated with lower CRP (β = -0.27 ±0.19, p<0.001) and lower fibrinogen (β = -13.5 ± 9.15, p=0.02). | 8 |
| Park, KH et al 2014  USA  (85) | 151 (53%)  Mean ~45 yrs | 110 item FFQ  AHEI  aMED | CRP | A, BMI, ETH, OBM, SX | Multivariate regression | Higher AHEI scores were associated with lower CRP (β = -0.02 ± 0.007, p=0.008)  Higher aMED scores were associated with lower CRP (β = -0.10 ± 0.04, p=0.02) | 8 |
| Rashidipour-Fard, N et al  2017  Iran  (86) | 107  >60 yrs | 70 item FFQ  HEI | CRP  Fibrinogen | A, BMI, EI, SX, WC | Multivariate linear regression | Higher HEI score was associated with lower CRP (β = -0. 74 (-0.386, -0.005), p=0.041) | 7 |
| Schæbel, LH et al  2013  Greenland  (87) | 535 (42%)  50-69 yrs | FFQ  IFIS | CRP | A, ALC, BMI, ETH, ODP, SX, SM | Multivariate linear regression | Higher IFIS was associated with higher CRP (p<0.001) | 9 |
| Schæbel, LH et al  2015  Greenland  (88) | 535 (42%)  50-69 yrs | FFQ  Greenlandic | CRP | - | ANOVA for between three groups of adherence to the diet. 100%->60%, 60%-40%, <40% | Higher adherence to the Greenlandic diet was associated with higher CRP. 100%->60% (1.6 (1.1-4.0), 60%-40% (1.4 (0.7-3.5)), <40% (1.3 (0.5, 2.3)). | 9 |
| Shivappa, N et al 2015  Belgium  Asklepios study (117) | 2,524  (52%) 35-55 yrs | 25-item SFFQ  DII | CRP IL-6  Fibrinogen | A, AHT, AID, BMI, EDU, EI, OCP, PA, SBP, SM, STA, SX | Multivariable logistic regression | Higher DII scores were associated with higher IL-6, (OR 1·19, (1·04, 1·36) | 8 |
| Sureda, A et al  2018 Spain  (91) | 234 (69%)  18-65 yrs | 2 x 24 hr diet recalls and 145 item FFQ  MDS | CRP  TNF-α Adiponectin | - | ANOVA for between groups above or below mean of MDS.  Stratified by sex | Men: Higher MDS score was associated with a higher adiponectin (13.1 ± 6.7, 9.5 ± 2.4, p<0.05) and lower TNF-α (7.9 ± 2.4, 12.3 ± 3.0, p<0.05) and CRP (0.17 ± 0.18, 0.41 ± 0.42, p<0.05).  Women: Higher MDS score was associated with a lower CRP (0.18 ± 0.23, 0.28 ± 0.32, p<0.05). | 5 |
| Tabung FT et al  2017  USA Nurses’ Health Study II (women) & the Health Professionals Follow-Up Study (men) (101) | 11,053  (53%) mean approx. 45 yrs | SFFQ  EDIP  DII | CRP IL-6 TNF-α Adiponectin | A, AID, CDCS, HRT, MPS, PA, SM | Multivariate linear regression | Women: Compared to the lowest the highest quintile of EDIP was associated with higher CRP (+50% (+25%, +79%), p<0.0001), IL-6 (+19% (+8%, +32%), p<0.0001) and TNF-α (+6% (+3% to +10%), p<0.0001) and lower adiponectin (-21% (-26% to -15%), p<0.0001). Compared to the lowest the highest quintile of DII was associated with higher CRP (+31% (+10%, +57%), p<0.0001), IL-6 (+15% (+3%, +27%), p=0.002), TNF-α (+3% (-1% to +6%), p=0.04) and lower adiponectin (-4% (-10% to +3%), p=0.009)  Men: Compared to the lowest the highest quintile of EDIP was associated with higher CRP (+33% (+19%, +49%), p<0.0001), IL-6 (+10% (-1%, +22%), p=0.04) and TNF-α (+8% (+5% to +12%), p<0.0001) and lower adiponectin (-16% (-20% to -11%), p<0.0001). Compared to the lowest the highest quintile of DII was associated with higher CRP (+20% (+19%, +49%), p<0.0001), IL-6 (+17% (+6%, +30%), p<0.0001) and TNF-α (+5% (+1% to +8%), p=0.002). | 7 |
| Tabung, F et al  2016  USA Nurses’ Health Study (women), Nurses’ Health Study II (women) & the Health Professionals Follow-Up Study (men) (93) | Nurses’ Health Study: 5230 (100%)  20-55 yrs  Nurses’ Health Study II:  1002 (100%) 25-42 yrs  Health Professionals Follow-Up Study:  2532 (0%) 40-74 yrs | SFFQ  EDII | IL-6 CRP TNF-α  Adiponectin | A, AID, CDCS, HRT, MPS, PA, SM | Multivariate linear regression | Nurses’ Health Study: Highest quintile of DII associated with higher IL-6 (β = 1.36, (1.28, 1.45), p<0.001), CRP (β = 1.82, (1.65, 2.01), p<0.001), TNF-α (β = 1.13, (1.10, 1.16), p<0.001)  Nurses’ Health Study II: Highest quintile of DII associated with higher IL-6 (β = 1.17, (1.02, 1.34), p=0.001), CRP (β = 1.52, (1.18, 1.97), p=0.002), TNF-α (β = 1.09, (1.04, 1.14), p=0.003) and lower adiponectin (β = 0.88 (0.80, 0.96), p=0.003)  Health Professionals Follow-Up Study: Highest quintile of DII associated with higher IL-6 (β = 1.14, (1.04, 1.24), p=0.01), CRP (β = 1.23 (1.09, 1.40, p=0.002), TNF-α (β = 1.07 (1.04, 1.10, p=0.001) and lower adiponectin (β = 0.87 (0.82, 0.92, p=0.003) | 7 |
| van Woudenbergh, GJ et al  2013  Netherlands  Diabetes and Atherosclerosis Maastricht (CODAM)  (94) | 1024 (46%)  mean ~64 yrs | FFQ  ADII | CRP  IL-6  TNF-α | A, BMI, CHT, FHD, HYP, PA, SM, STA, SX | Multivariate liner regression | Higher ADII scores were associated with higher IL-6 (β = 0.04 (0.01, 0.08, p=0.02). | 7 |
| Villegas, R et al  2012  China  Shanghai Men’s Health Study (SMHS)  (95) | 3,978 (0%)  40-74 yrs | FFQ  FA-(legumes & vegetables, fruit, meat) | CRP | A, ALC, BMI, CVD, EDU, EI, INC, OCC, PA, SM, TEA | Logistic regression for CRP ≤3mg/L and >3mg/L and CRP | Higher adherence to the fruit pattern was associated with lower CRP (OR 0.68 (0.46, 0.99), p=0.04). | 5 |
| Viscogliosi, G et al  2013  Italy  (96) | 120 (64%)  mean 60 yrs | 14-item questionnaire  MDP | CRP | EDU, FHD, MED, MS, PA, SM, WC, WS | Multivariate linear regression | Higher MDP scores were associated with lower CRP (β = -0.082, (-0.125, -0.045), p<0.0001) | 8 |
| Whalen, KA et al  2016  USA  (97) | 646 (69%)  30-74 yrs | FFQ  PLD  MedDietScore | CRP | A, AID, EDU, EI, HRT, NSAID, PA, SEA, SM, SMI, SX, VIT | Logistic regression | Higher MedDietScore was associated with lower CRP (OR 0.71 (0.42, 1.2), p=0.01) | 7 |
| Wirth, M et al  2014  USA  Buffalo Cardio-Metabolic Occupational Police Stress study  (98) | 447 (25%)  mean 42 yrs | FFQ  DII | CRP  IL-6  TNF-α | CRP: A, EDU  IL-6: ALC, RNK, YPW  TNF-α: A, ETH, SX | GLM | No association found. | 6 |
| Xu, H et al  2015  Sweden Uppsala Longitudinal Study of Adult Men (ULSAM) and the Prospective Investigation of Vasculature in Uppsala Seniors (PIVUS) (118) | 1,942 (22%) 70-71 yrs | 7-day food record  ADII | CRP | A, CHT, DIA, HYP, PA, SM, STA, SX | Linear regression | Higher ADII were associated with higher CRP levels (β = 0.06, (0.01, 0.10), p=0.01) | 6 |
| Yannakoulia, M et al  2008  Greece  (100) | 220 (100%)  mean 47 yrs | 3-day food record  PCA-( vegetables, fruits & olive oil; whole-grain cereals & low-fat dairy & low in refined cereals; coffee & low in low-fat dairy; full-fat dairy; poultry & low in red meat; fish & low in red meat; high alcoholic beverages; nuts & low in fruits; legumes; potatoes) | Adiponectin | BMI, EMR, MPS, ODP, PA | Multivariate linear regression | Higher adherence to the vegetables, fruits & olive oil pattern was associated with higher adiponectin (β = 0.18, p=0.03) | 6 |

| *Longitudinal studies* | | | | | | | |
| --- | --- | --- | --- | --- | --- | --- | --- |
| Authors, Year, Country, Cohort, Follow-up period, Reference | **Sample size, (% women),**  **Age** | **Diet collection method,**  **Diet assessment** | **Outcome variable/s** | **Confounders examined** | **Statistical methodology** | **Results, Odds ratio or β coefficient, 95% confidence intervals, p-values** | **Score** |
| Akbaraly T N et al 2015 UK Whitehall II 6 yrs follow-up (119) | 4600  (28%) mean 49.6 yrs | 127 item FFQ  AHEI | IL-6 CRP | A, AID, BMI, CHD, DIAB, DOM, ETH, EI, HDL, HYP, PA, SES, SM, SX | % difference (exp [linear regression coefficient] -1) *100 | High AHEI or moving from low to high AHEI score associated with lower mean levels of IL-6 (-8.6% (± 1.6), (-8.1% (± 2.1). | 13 |
| Cavicchia, PP et al  2009  USA  Seasonal Variation of Blood Cholesterol Study (SEASONS)  1 yr  (39) | 494 (47%) mean 48 yrs | 3 x 24 dietary recalls collected quarterly  II | CRP | AID, EDU, EI, ETH, HSP, LS, PA, SP, SX, TC | Multivariate linear regression | No associations found | 13 |
| Fargnoli, JL et al 2008 USA  6 yrs  Nurses’ Health Study  (48) | 1922 (100%) mean ~56 yrs | SFFQ AHEI | Adiponectin IL-6 CRP TNF-α | A, BMI, EI, PA, SM | Multiple linear regression | Higher AHEI scores were associated with higher adiponectin (p=0.0004), lower CRP (p=0.02). | 11 |
| Jacobs, S et al  2017  USA  Multiethnic Cohort  9.5 years  (63) | 10,060 (54%  Mean ~58 yrs | QFFQ HEI, aHEI, aMED, DASH | CRP  Adiponectin | A, BMI, EDU, EI, ETH, PA, SM | GLM | Higher DASH scores were associated with higher adiponectin in men (p<0.05) and women (p<0.05).  Higher aMED and DASH scores were associated with lower CRP in men (p<0.05) and women (p<0.05).  Higher HEI and aHEI scores were associated with lower CRP in men only (p<0.05). | 11 |
| Julia, C et al  2017  France  12 yrs SUpplémentation en VItamines et Minéraux AntioXydants (SU.VI.MAX) 2 study  (65) | 1980 (54%)  mean 50 yrs | Repeated 24-hr diet record  DII, aDII | CRP | A, EDU, EI, NDR, PA, SM, SPG, SX | Multivariate logistic regression | No associations found. | 11 |
| Julia, C et al  2013  France  12 yrs  SUpplémentation en VItamines et Minéraux AntioXydants (SU.VI.MAX) 2 study  (66) | 2031 (53%)  ~50 yrs | Repeated 24-hr diet record  RRR-(DP1-vegetables, olive oil & other vegetable oils, DP2- fatty fish, seafood, processed meat, organ meat, eggs & poultry, DP3- fruits & fruit juices, DP4- processed meat, eggs and poultry) | CRP | A, BMI, EDU, MS, NDR, PA, SM, SPG, SX | Logistic regression for diet patterns and CRP ≤3 or >3 mg/l | Higher adherence to DB1 was associated with reduced odds of elevated CRP (OR 0.88 (0.78, 0.98), p = 0.025). Higher adherence to DP4 was associated with higher odds of elevated CRP (OR 1.15 (1.00, 1.32) p = 0.046) | 11 |
| Mattei, J et al  2017  USA 2 yrs follow-up  Boston Puerto Rican Health Study (76) | 1,194  45-75 yrs | SFFQ  AHEI, AHADS, DASH, HEI, MDS | CRP | A, AID, CDV, DIA, EDU, GLU, HYP, INC, MS, PA, SM, SX | Multivariable-adjusted, repeated-subjects, linear mixed-effects models for diet at baseline and CRP at 2 yrs | Higher MDS was associated with lower CRP (β = -0.13 ±0.03, p=0.0002) | 9 |
| Mertens, E et al  2018  UK  11.8 yrs  Caerphilly Prospective Study (CaPS)  (77) | 766 (0%)  45-59 yrs | SFFQ  AHEI-2010  DASH  HDI | CRP | A, ALC, BMI, EI, PA, SES, SM | Multivariate linear regression | Higher AHEI-2010 scores were associated with and 11% decrease in CRP (p<0.005) | 11 |
| Shivappa N et al  2014 USA  Seasonal Variation of Blood Cholesterol Study (SEASONS) 1 yr follow-up (90) | 1054 (47%) 20-70 yrs | 3 x 24hr food recall or 7 DDR  DII | CRP | A, AID, ALC, EDU, ES, ETH, HDL, HSP, LS, MS, PA, SM, TC | Multivariable logistic regression | Higher DII scores were associated with higher CRP, 24 hr recall (OR = 1.08, (1.01, 1.16), p=0.035) 7DDR (OR = 1.10; (1.02, 1.19), p=0.015) | 7 |

**Diet**: ADII-Adapted Dietary Inflammatory Index, AHADS-American Heart Association Diet Score, AHEI-Alternative Healthy Eating Index, AIDA-Anti-Inflammatory Diet Index, aMED-alternate Mediterranean Diet Score, BDHQ-Brief self-administered diet history questionnaire, BSDS- Baltic Sea Diet Score, CA- Cluster analysis, CDS-Composite Diet Score, CHDP- Comprehensive Healthy Dietary Pattern, DASH-Dietary Approaches to Stop Hypertension, DDS-R Dietary diversity score for recommended foods, DED-Dietary Energy Density, DII-Dietary Inflammatory Index, DQI-I- Diet Quality Index-International, DQI-R-Diet Quality Index Revised, DQI-SNR-Diet Quality Index- Swedish Nutrition Recommendations, EDII-Empirically Derived Dietary Inflammatory Index, EDIP- Empirical Dietary Inflammatory Pattern, FA-Factor analysis, FBDG-Food-based dietary guidelines, FFQ-Food Frequency Questionnaire, IFIS- Inuit food item scores, IFIS- Inuit food item score, II-Inflammatory Index, HDI-Healthy diet indicator, MedDietScore-Mediterranean Diet Score, MDS-Mediterranean Diet Score, MDP-Med Diet Pattern, PLD-Palaeolithic Diet, PCA-Principal component analysis, QFFQ-Quantitative Food Frequency Questionnaire, RCI-Recommended Compliance Index, SHDP- Simplified Healthy Dietary Pattern, SFFQ-Semi-quantitative Food Frequency Questionnaire, TDP-Taiwanese Dietary Pattern

**Outcomes**: CRP-C-reactive protein, IL-6-Interleukin 6, TNF-α-Tumour Necrosis Factor-alpha

**Confounders**: A-Age, AFB- Age of first birth (women), ALC-Alcohol intake, AID-Anti-inflammatory Drugs, ASP-Aspirin use, BMI-Body Mass Index, BPM-Blood Pressure Medication, CAN-Cancer status, CDS- chronic disease comorbidity score, CHD-Coronary Heart Disease, CHT-Cohort, CON- Country; CS-Clinical site, DBM-Diabetes medication, DBP-Diastolic Blood Pressure, DIAB-Diabetes, DOM-Domestic living , DPFS-Dietary pattern factor scores, DS-Depressive Symptoms, EDU-Education level, EGG- egg consumption, EI-Total energy intake, EMR-Energy misreporting; ES-Employment Status, ETH-Ethnicity, FHM-Family history of myocardial infarction, FHD-Family history of diabetes, FM-Fat body mass, FOS-Fish oil supplementation, GLU-Plasma glucose level, HC-Hypercholesterolemia, HDL-High Density Lipoprotein, HRT-Hormone Replacement Therapy, HSP- herbal supplements, HYP-Hypertension, INC-Income, IQ-Intelligence Quotient Score, LDL-Low-density lipoprotein, LM-Lean body mass, LS-Light season, MED-medications, MPS-Menopause, MRB-Morbidity, MS-Marital Status, NDR-Number of dietary records, NSAID- nonsteroidal anti-inflammatory drug use, OBM-Other biomarkers, OCC-Occupation, OCT-Oral Contraceptive Pill, ODP-Other dietary patterns, PA-Physical Activity, PBF-Percentage body fat, POT-Potato consumption, PRO-Protein intake, RNK-Rank, SBP-Systolic Blood Pressure, SC-Study Centre, SEA-Season, SES -Socio-economic Status, SM-Smoking Status, SP-Sleep duration, SPG-Supplementation/placebo group, STA-Statin use, SX-Sex, TEA-Tea consumption, TC-Total Cholesterol, TRI-Triglycerides, TV-Television watching time, VIT-Vitamin use, WC-Waist Circumference, WHR-Waist to hip ratio, YPW-Years of police work, ZY-Zygosity

yrs- years SE-Standard error
